# Persistent *Chlamydophila pneumoniae* infection in thoracic aortic aneurysm and aortic dissection?

**DOI:** 10.3109/03009731003778719

**Published:** 2010-07-19

**Authors:** Marie Edvinsson, Stefan Thelin, Eva Hjelm, Göran Friman, Christina Nyström-Rosander

**Affiliations:** ^1^Department of Medical Sciences, Infectious Diseases, Uppsala University Hospital, Uppsala University, UppsalaSweden; ^2^Department of Surgical Sciences, Thoracic Surgery, Uppsala University Hospital, Uppsala University, UppsalaSweden; ^3^Department of Medical Sciences, Clinical Bacteriology, Uppsala University Hospital, Uppsala University, UppsalaSweden

**Keywords:** Aortic dissection, Chlamydia pneumoniae, Chlamydophila pneumoniae, inflammation, thoracic aortic aneurysm

## Abstract

**Objectives:**

*Chlamydophila pneumoniae* (*C. pneumoniae*) has been associated with atherosclerosis and abdominal aortic aneurysm and is probably disseminated by peripheral blood mononuclear cells (PBMC). Viable and metabolically active bacteria can be demonstrated by the presence of bacterial mRNA and on-going dissemination by the presence of bacteria in PBMC. The aim of this study was to determine the prevalence of *C. pneumoniae* DNA and mRNA in aortic biopsies and *C. pneumoniae* DNA in PBMC in thoracic aortic aneurysm and aortic dissection patients.

**Design:**

Real-time PCR was used to detect *C. pneumoniae* DNA and mRNA in biopsies and *C. pneumoniae* DNA in PBMC.

**Results:**

*C. pneumoniae* DNA was found in biopsies in 26% (6/23) of aneurysm patients and 11% (2/18) of dissection patients but in none of the forensic autopsy controls. *C. pneumoniae* mRNA was not found in any biopsy, and all PBMC were *C. pneumoniae*-negative.

**Conclusions:**

Presence of *C. pneumoniae* DNA but not mRNA in aortic biopsies and no evidence of *C. pneumoniae* in PBMC suggest that the infection in the aorta has passed into a state of persistence.

## Introduction

Thoracic aortic aneurysm and aortic dissection are two major diseases affecting the thoracic aorta, both of which may cause aortic rupture. In an aortic aneurysm there is a dilatation of all three layers of the vessel wall, compared to aortic dissection where there is a splitting of the wall layers. The pathogenesis of most cases of thoracic aortic aneurysm and dissection is unknown, but in both diseases it is believed to involve medial degeneration leading to a weakening of the aortic wall (1). Known risk factors associated with thoracic aortic aneurysms are bicuspid aortic valve, Turner's syndrome, and aortic arteritis (e.g. Takayashu's arteritis and giant cell arteritis) (1). The most common associated risk factors for the development of aortic dissection are hypertension and advanced age; other risk factors are smoking, dyslipidemia, diabetes mellitus, and Turner's syndrome (2). Previously, a common cause of aneurysm of the ascending aorta was syphilis, which today, with declining incidence, is a rare cause.

In abdominal aortic aneurysm the main factor in the pathogenesis is believed to be atherosclerosis, in contrast to thoracic aortic aneurysm where atherosclerosis is found less often. The medial degeneration of thoracic aortic aneurysms has previously been described as a non-inflammatory disease; however, recent studies have demonstrated the presence of inflammatory cells in the aneurysm, indicating that inflammation may have a role in the pathogenesis of thoracic aortic aneurysms (3). Moreover, because gene expression studies have demonstrated up-regulated genes involved in inflammatory processes, inflammation may have a role also in aortic dissection (4).

The respiratory pathogen *Chlamydophila pneumoniae* (*C. pneumoniae*) has been suggested to be involved in the pathogenesis of atherosclerosis as an initiator and stimulator of the chronic inflammation in the atherosclerotic plaque (5). This obligate intracellular bacterium has a unique biphasic life cycle that sometimes may be interrupted so that the bacterium passes into a persistent state unsusceptible to antibiotics (6). This ability of *C. pneumoniae* to cause chronic disease makes it difficult to isolate from patient materials; in fact, only a few studies have succeeded in culturing *C. pneumoniae* from atherosclerotic tissue (7,8). On the other hand, many studies have demonstrated the presence of *C. pneumoniae* DNA within the atherosclerotic plaque (9); however, the presence of bacterial DNA does not indicate whether the bacterium is replicating and metabolically active or not. Viable and metabolically active *C. pneumoniae* may instead be proven by demonstration of bacterial mRNA in the tissue, which has been done in biopsies from the thoracic aorta in patients undergoing coronary artery by-pass surgery (CABG) (10). Dissemination of *C. pneumoniae* from the respiratory tract is believed to be mediated by alveolar macrophages, and in patients suffering from cardiovascular diseases *C. pneumoniae* has often been detected in peripheral blood mononuclear cells (PBMC) at a higher detection rate than in healthy blood donors (11).

We have previously demonstrated the presence of *C. pneumoniae* DNA in 12% of thoracic aortic aneurysm biopsies and in none of six aortic dissection biopsies (12). In a more recent study of patients undergoing thoracic surgery because of thoracic aortic aneurysm we have demonstrated the presence of *C. pneumoniae* DNA in 26% of the thoracic aortic aneurysms (13). Moreover, trace elements were measured in serum and aortic tissue in these patients, and changes indicating on-going infection/inflammation were demonstrated compared to healthy controls. In the present study we aimed to further characterize the state of the infection in these patients by investigating the presence of *C. pneumoniae* mRNA in aortic tissue as well as the presence of *C. pneumoniae* in PBMC. Furthermore, the presence of *C. pneumoniae* was also determined in patients undergoing thoracic surgery because of Type A aortic dissection.

## Material and methods

### Patients and patient samples

In the present study 25 patients undergoing thoracic surgery because of ascending thoracic aortic aneurysm and 21 patients undergoing surgery because of Type A aortic dissection were included. The aneurysm patient group consisted of 17 men and 8 women with a mean age of 61 years (range 30–81 years). The aortic dissection patient group had a mean age of 61 years (range 45–78 years) and consisted of 15 men and 6 women. Clinical data on patients are presented in Table I. None of the patients had a hereditary connective tissue disorder. Thoracic aortic wall biopsies were obtained from aneurysmatic or dissecting tissue during surgery and aseptically divided. One part of the biopsy was placed in RNAlater (Qiagen, Solna, Sweden) overnight and then frozen at −70°C, and another was fixed in 10% formalin solution. The remaining parts of the biopsy were immediately frozen at −70°C until further processing. Blood samples were collected for antibody and for preparation of PBMC. Sera were frozen at −20°C until further analysis. Throat and nasopharyngeal swabs for *C. pneumoniae* detection were collected using CTA swabs transported in Tris-buffer (pH 7.0) and then frozen at −70°C until further analysis. For technical reasons, biopsy specimens for DNA analyses were not obtained from two aneurysm and three dissection patients and not for mRNA analyses from one aneurysm and two dissection patients.

**Table I.** Clinical data on thoracic aortic aneurysm (*n* = 25) and aortic dissection patients (*n* = 21).

	Aneurysm	Dissection
Arterial hypertension	44% (11/25)	71% (15/21)
Hyperlipidemia	28% (7/25)	14% (3/21)
Diabetes mellitus	4% (1/25)^a^	0% (0/21)
Smoker/former smoker	52% (13/25)	43% (9/21)
Angina pectoris	20% (5/25)	14% (3/21)
Previous myocardial infarction	4% (1/25)	5% (1/21)
Undergoing CABG in same operation	16% (4/25)	14% (3/21)
Undergoing aortic valve replacement in same operation	60% (15/25)	10% (2/21)
Normal coronary arteries verified by coronary angiography	74% (17/23)^b^	nd
Bicuspid aortic valve	32% (8/25)	nd
Family history of cardiovascular disease	20% (5/25)	5% (1/21)

^a^The one patient with diabetes mellitus had Type 1 diabetes mellitus.

^b^Angiography was not performed on two patients. CABG = coronary artery by-pass surgery; nd = not determined.

### Controls and control samples

Control specimens of thoracic aorta were collected from 10 forensic autopsy controls consisting of 6 women and 4 men (mean age 61 years, range 41–80 years) without known cardiovascular disease. Biopsies were aseptically divided and frozen at −70°C until analysis of *C. pneumoniae* DNA.

### Preparation of PBMC

PBMC was isolated from 4 mL whole blood collected in heparinized tubes. Blood samples were diluted with an equal volume of phosphate buffered saline (PBS) and layered onto Ficoll-Paque PLUS (Amersham Biosciences, Uppsala, Sweden). Tubes were centrifuged for 30 minutes at 400 *g*, and the buffy-coat layer was collected and washed with an equal volume of PBS twice for 10 minutes at 100 *g*. Remaining cells were suspended in approximately 0.5 mL of saline solution and frozen at −70°C.

### DNA extraction

DNA was extracted from approximately 10 mg tissue, throat and nasopharyngeal swabs, and 300 μL suspended PBMC using the QiaAmp DNA mini kit (Qiagen) according to instructions from the manufacturer and then eluted in 50 μL buffer AE. In every round of extraction a negative no-template control was processed in the same way as the samples.

### RNA extraction

RNA was extracted from approximately 25 mg tissue sample treated with RNAlater using the RNeasy fibrous tissue mini kit (Qiagen) according to instructions from the manufacturer and then eluted in 30 μL RNase-free water. Aneurysm samples were homogenized in Lysing Matrix A tubes (In Vitro AB, Stockholm, Sweden) on a Fast Prep FP120 (In Vitro AB, Stockholm, Sweden). Dissection samples were homogenized in a TissueLyser (Qiagen). RNA concentration in samples was measured on a NanoDrop ND-1000 (NanoDrop Technologies Inc., Wilmington, DE, USA) and subsequently stored at −70°C until further processing.

### Reverse transcription PCR

cDNA was synthesized from RNA using the SuperScript First-Strand Synthesis System for RT-PCR (Invitrogen, Stockholm, Sweden) with random hexamers according to instructions from the manufacturer. In every RT-PCR reaction 14 μL of extracted RNA was used.

### Real-time PCR

DNA and cDNA samples were subjected to real-time PCR, amplifying a fragment of the *C. pneumoniae ompA* gene as has previously been described (10). Control samples of a known concentration were included in each run to verify PCR reproducibility by checking for differences in Ct values. To verify that DNA extraction had been accomplished, PCR against the human beta-actin gene was run on all DNA samples (14). Further, to verify that mRNA had not been degraded during handling, all cDNA samples were tested for the presence of human glyceraldehyde-3-phosphate dehydrogenase (GAPDH) transcripts using TaqMan GAPDH control reagents (Applied Biosystems, Stockholm, Sweden) according to the manufacturer's instructions. In these reactions 5 μL of cDNA diluted 1:10 was used. To check for possible PCR inhibition, several negative DNA samples were spiked with *C. pneumoniae* DNA, and real-time PCR was run.

### Serology

Patient sera were tested for *C. pneumoniae*-specific IgM, IgG, and IgA antibodies by the micro-immunofluorescence method using microscopic slides from ANI Labsystems OY (Helsinki, Finland) (10).

### Light microscopy

Biopsies were fixed in 10% formalin solution. They were embedded in paraffin, sectioned, and stained with May Grünwald Giemsa (MGG), Alcian Blue, van Gieson, Elastin (AVE), and Grocott stain.

### Ethics

The Research Ethics Committee of the Faculty of Medicine Uppsala University, Uppsala, approved this study (D. No. 03 244). Informed consent was obtained from all patients, and the investigation conformed to the principles outlined in the Declaration of Helsinki.

## Results

### Real-time PCR

Of the biopsy samples, 6/23 (26%) aneurysm and 2/18 (11%) dissection patient samples were positive for *C. pneumoniae* DNA but none of the forensic control specimens were. Concentrations of *C. pneumoniae* in the positive samples were low, with only a few copies of *C. pneumoniae* per mg tissue. In the PBMC all patients tested negative for *C. pneumoniae* DNA. Throat swabs were positive in 2/25 aneurysm patients but in none of the 21 dissection patients. Nasopharyngeal swabs were positive in 1/21 dissection patients but in none of the 25 aneurysm patients. Those three positive patients were only positive in the nasopharynx or throat and not in the aorta. Moreover, all these samples contained few copies of *C. pneumoniae*. All aortic samples tested negative for *C. pneumoniae* mRNA. Human GAPDH mRNA was found in all cDNA, indicating that mRNA had not been degraded. All DNA samples tested positive for the presence of human beta-actin. Spiked samples showed no signs of inhibition. The present results of *C. pneumoniae* DNA in thoracic aortic aneurysm biopsies and in forensic control specimens have previously been published (13).

### Serology

IgG antibodies ≥1:64 against *C. pneumoniae* were found in 56% (14/25) of the aneurysm patients, in 43% (9/21) of the dissection patients, in 83% (5/6) of the aneurysm patients positive for *C. pneumoniae* in the aorta, and in 50% (1/2) of the dissection patients positive for *C. pneumoniae* in the aorta. Mean IgG titre was 1:123 in the aneurysm patients and 1:67 in the dissection patients. IgA antibodies ≥1:16 were found in 28% (7/25) of the aneurysm patients with a mean titre of 1:24. None of the dissection patients had any IgA antibodies against *C. pneumoniae*.

### Histopathology

Samples for histopathological analyses were obtained from 23 thoracic aortic aneurysm patients and 17 aortic dissection patients. Among the eight patients positive for *C. pneumoniae* in the aorta, samples for histopathological analyses were obtained from 5/6 aneurysm patients and from both dissection patients. Cystic media necrosis, atherosclerotic changes, and inflammatory cells in the biopsies are shown in Table II.

**Table II.** Histopathological analyses in aortic biopsies from thoracic aortic aneurysm and aortic dissection patients.

	Thoracic aortic aneurysm patients (*n* = 23)		Aortic dissection patients (*n* = 17)	
	*C. pneumoniae*-positive (*n* = 5)	*C. pneumoniae*-negative (*n* = 18)	*C. pneumoniae*-positive (*n* = 2)	*C. pneumoniae*-negative (*n* = 15)
Cystic medial necrosis	1/5	1/18	0/2	4/15
Atherosclerotic changes	1/5	8/18	1/2	1/15
Inflammatory cells	5/5	8/18 ^a^	0/2	8/15
Atherosclerotic changes and inflammatory cells	1/5	4/18	0/2	0/15
Cystic medial necrosis and inflammatory cells	1/5	0/18	0/2	1/15

^a^*P* < 0.05, difference between *Chlamydophila pneumoniae*-positive and *C. pneumoniae*-negative aneurysm.

## Discussion

*Chlamydophila pneumoniae* (*C. pneumoniae*) DNA was found in thoracic aortic biopsies from 26% (6/23) of the patients undergoing surgery for thoracic aortic aneurysm and from 11% (2/18) of the patients with aortic dissection. *C. pneumoniae*-specific mRNA, a marker of metabolically active bacteria, could not be found in any of the biopsies, and none of the patients had *C. pneumoniae* in their PBMC. This may indicate that the acute phase of the *C. pneumoniae* infection occurred long before the patient was undergoing surgery and that the infection in the aortic wall had passed into a state of persistence.

*C. pneumoniae* has been demonstrated by PCR in abdominal aortic aneurysm in several studies (15,16), and viable bacteria have been cultured from aneurysmatic tissue (7). We have previously demonstrated *C. pneumoniae* DNA in 12% of thoracic aortic aneurysm and in 0% of aortic dissection tissue (12), whereas another group of investigators failed to demonstrate the presence of *C. pneumoniae* in this kind of tissue, as well as in abdominal aortic aneurysm (17). Furthermore, another study failed to demonstrate the presence of *C. pneumoniae* in Type A aortic dissection tissue (18). In the present study *C. pneumoniae* DNA was found in 26% of the thoracic aortic aneurysm patients (13) and in 11% of the aortic dissection patients but in none of the control aortic biopsies. The higher prevalence in this new study than in our previous one (12) may be due partly to the use of a more sensitive PCR method and partly to a potential patchy distribution of the bacteria (5).

In contrast to abdominal aortic aneurysm, atherosclerosis is believed to be an infrequent cause of thoracic aortic aneurysm and of aortic dissection (19). The aneurysm patients in this study had a rather low degree of coronary artery stenosis, as verified by coronary angiography. On the other hand, atherosclerotic changes in the aneurysm were demonstrated by histology in 9/23 patients; however, these changes were not more prevalent in *C. pneumoniae*-positive patients than in *C. pneumoniae*-negative patients. Regarding presence of inflammatory cells, no difference was noted between aneurysm and dissection biopsies. However, among the aneurysm biopsies, inflammatory cells were significantly more frequent in *C. pneumoniae*-positive aneurysms than in *C. pneumoniae*-negative aneurysms. No such difference existed among the dissection biopsies.

Presence of *C. pneumoniae* in circulating PBMC may be associated with cardiovascular disease (11). However, detection rates in different studies on such patients vary between 0% (20) and 59%, and detection rates in healthy blood donors vary from 0% to 46% (11). Such wide variation can probably be explained by differences in sampling, nucleic acid extraction methods, and PCR techniques. To our knowledge, this is the first study of *C. pneumoniae* in PBMC of thoracic aortic aneurysm. One study has investigated the presence of *C. pneumoniae* in PBMC in aortic dissection patients, and all patients with Type A aortic dissection were negative for *C. pneumoniae* DNA (18). All patients in this study tested negative for *C. pneumoniae* in PBMC, indicating that the bacteria probably had disseminated to the aorta long before the patient was in need of surgery.

Isolation of *C. pneumoniae* from atherosclerotic tissue has only been successful in a few studies (7,16), and then only after several passages. This work is time-consuming, with a high risk of contamination. Instead, viable and metabolically active *C. pneumoniae* can be demonstrated by the presence of bacterial mRNA. We have previously shown that patients undergoing CABG have *C. pneumoniae* mRNA coding for the major outer membrane protein in macroscopically normal thoracic aorta (10). However, in that study samples that were positive for *C. pneumoniae* DNA were not always positive for *C. pneumoniae* mRNA. Most of the aortic samples in the present study were altered by advanced disease, including fibrosis and calcification, and they were all negative for *C. pneumoniae* mRNA. All samples contained rather low amounts of RNA but were, however, positive for human cDNA, indicating that the mRNA extraction had worked. This suggests that the bacteria have entered into a persistent and non-active state in the disease-altered tissue and are thus not producing measurable amounts of mRNA.

*C. pneumoniae* antibodies were determined with the micro-immunofluorescence method. Considering the age of the patients, antibody titres seemed to be in the normal range (6), with 56% of the aneurysm patients and 43% of the dissection patients having IgG against *C. pneumoniae*.

Inflammatory cells have previously been demonstrated in thoracic aortic aneurysms (21), and it has been suggested that different antigens may be involved in the formation of the aneurysms (3). These antigens could possibly be micro-organisms, as we have demonstrated the presence of *C. pneumoniae* in the aneurysms and others have demonstrated other bacteria in the aneurysms (22). Moreover, *in vitro* studies have demonstrated that *C. pneumoniae* is able to degrade elastin, a load-bearing protein in the aortic wall (23), and these mechanisms may contribute to the pathogenesis of the rupture of the aortic wall.

Certain changes in trace element concentrations can be used as markers of infection/inflammation (24). We have previously demonstrated such markers in sera of patients undergoing surgery because of aortic valve stenosis, where a large number of the patients were positive for *C. pneumoniae* in the valve (25). In a more recent study of sera and aortic biopsies of the thoracic aortic aneurysm patients in the present study we demonstrated trace element changes, indicating on-going infection/inflammation in the patients compared to healthy controls (13).

In conclusion, the results in the present study demonstrate that *C. pneumoniae* may be found in the aortic wall in thoracic aortic aneurysm patients and aortic dissection patients though not in a metabolically active state. Dissemination of *C. pneumoniae* from the respiratory tract to the aortic wall may have occurred long before the patient was in need of thoracic surgery as no *C. pneumoniae* could be found in the PBMC. This indicates that the *C. pneumoniae* infection in the patients is not in an active state but has probably passed into a chronic state of persistence. Further studies are needed to establish the role of inflammation, which is possibly caused in some patients by slow-growing bacteria, including *C. pneumoniae*, in the pathogenesis of thoracic aortic aneurysm and aortic dissection.
